# Supplementary material for: Are carbon emissions trading and green financial instruments synergistic? -Comprehensive quantitative research based on content analysis
Source: PLoS One. 2024 Mar 7;19(3):e0298601. doi: 10.1371/journal.pone.0298601 (PMC10919601; doi:10.1371/journal.pone.0298601)
Supplement: S1 Data — (ZIP) [file pone.0298601.s001.zip › Data/地级市Z_F工作报告.zip!/╔┬╬≈/╤╙░▓╩╨/└┤╫╘╣1⁄2╓┌║┼í╛┬φ┐╦╩2╛▌═°í┐_2018.docx]

各位代表：

现在，我代表市人民政府向大会报告工作，请予审议，并请市政协委员和其他列席人员提出意见。

一、市五届人大一次会议确定任务完成情况

2017年，是延安转型发展、追赶超越进程中极不平凡的一年。在市委的坚强领导下，我们以习近平新时代中国特色社会主义思想为指导，深入贯彻新发展理念，全力推进“转型升级十大产业项目”、“基础设施十大建设工程”和“人民幸福十件实事”，经济社会发展态势良好，实现本届政府工作的良好开局。完成生产总值1266.4亿元，增长7.6%，较上年提高6.3个百分点，是2013年以来最好水平；地方财政收入140.4亿元，增长7.6%，较上年提高26.6个百分点；城乡居民人均可支配收入分别达到33168元和11525元，均高于全省平均水平。

（一）经济保持平稳较快增长。一产规模质量效益稳步提升，增加值增长5%，居全省第二位。建成市级“一村一品、一乡一业”专业村255个、专业镇23个。建成市级现代农业示范园12个，累计达到62个。生产粮食76.7万吨。新建果园12.1万亩，苹果总产323.2万吨。生产蔬菜138.5万吨。发展规模化养殖场391个。全力稳定工业增长基本面，制定《工业稳增长促投资12条措施》，生产原油1516.5万吨，与上年持平，加工原油907.8万吨、下降2.1%，生产煤炭5015.2万吨、增长10%，生产天然气39.5亿立方米、增长17.1%，LNG产量82.9万吨、增长8%，发电42.6亿千瓦时、增长10.8%。高端卷烟产品占比提升，“延安”青春岁月卷烟成功上市，生产卷烟35.1万箱，产值增长实现扭负为正。努力提升服务业发展水平，三产增加值增长10%，居全省第二位。新增规模以上服务业企业47户、规模以上文化企业12户。22个市级重点文化旅游项目进展顺利。南泥湾景区开发全面启动，梁家河数字博物馆上线，延安国际滑雪场等旅游项目建成运营。荣获2017“十佳魅力城市”和“十大文化影响力城市”。接待游客突破5000万人次，旅游综合收入近300亿元。红色教育培训人数增长65.7%。中国供销总社农产品物流园区一期建成。金融机构贷款余额首次突破1100亿元，存贷比为70.9%。注册电子商务经营企业1193户，网店微店突破2万家。社会消费品零售总额增长10.4%。进出口贸易总额增长22.1%。

（二）结构调整实现重大突破。持续推进高端能化，富县煤油气资源综合利用项目实现中交，洛川轻烃裂解综合利用、延长石油伴生气资源循环利用、子长500万吨低阶煤多联产循环综合利用项目进展顺利。《延安综合能源基地发展规划》获得国家能源局批准，陕北至关中750千伏第二通道开工建设，延安至西柏坡电力外送通道前期工作和延安新区、安塞区多能互补项目加快推进，8个新能源项目并网发电。加速培育增长新动能，新区创8空间、基金小镇、高新区“红色筑梦”科技孵化器、宝塔区北航贝塔等4家众创空间正式运营，英雄互娱、国创科视等201家新经济企业落地，注册资本410多亿元，实现营业收入15亿元。高新区创新引领能力明显增强，珠海兴业新材料项目建成投产，大唐热电联产项目建成并向市区供热，新加坡三达膜科技、石墨烯科技产业园等高新技术产业项目落地。17个县域工业集中区新增入园企业42户，营业收入增长15%。新登记市场主体2.9万户，新增“五上”企业159户。非公经济增加值增速和占GDP比重增幅均居全省第一。强力推进以苹果为主的农产品后整理，建成智能选果线6条，新增冷气库储能10.5万吨，发展农产品加工企业746家。洛川县、富县荣获“国家农产品质量安全县”称号，富县成功创建全国良好农业规范认证示范县。“延安苹果”、“黄陵翡翠梨”成功注册国家地理标志证明商标，新认证“三品一标”农产品93个，欧盟有机认证20个。

（三）重大项目建设取得明显进展。市级重点项目开复工率创近年新高。“三个十大项目”开复工率达到100%。完成全社会固定资产投资1283.7亿元，下降5.6%，降幅较上年收窄9.8个百分点。沿黄公路建成通车，引黄工程具备向市区供水条件，延安新机场、西延高铁、蒙华铁路等项目进展顺利，吴起至定边、绥德至延川、清涧至子长等8条通县高速公路加快推进。新增延安至青岛、沈阳、杭州、南京等城市航线。建成吉现330千伏输变电站。百兆光纤宽带进村入户。全面实施延河综合治理、山水林田湖草生态修复试点。我市被确定为国家重大市政工程领域PPP创新工作重点城市，25个PPP项目取得实质性进展。规模300亿元的延安振兴发展产业投资基金进入募投阶段。

（四）新型城镇化步伐加快。我市被国土资源部列为新一轮土地利用总体规划（2020-2035年）试点。《延安城市总体规划（2015-2030）》修编获得省政府批复。住建部“城市双修”和城市设计试点工作全面展开，实施城改项目56个，城区棚户区改造累计征收动迁16.1万人。城区综合管廊试验段主体完工，延安植物园一期和2条城市道路环线建成，新建改造便民市场15个，硬化巷道20条，新建公园广场14个、停车场（楼）10个。免费开放城区203个停车场、5596个停车位。新区建设助推老城功能疏解，城市主干路网基本形成，2所学校和5所幼儿园建成，学习书院成为学习习近平新时代中国特色社会主义思想的新阵地，华为云计算数据中心、金融服务中心、北大阳光教育小镇等项目加快推进，保障房、商品房陆续入住，朝气蓬勃的现代化新城正在崛起。市级城市管理执法体制改革受到住建部表彰。“2+1”三城联创带动城市品位和市民素质显著提升，荣获国家卫生城市、国家园林城市称号，成功创建省级节水型城市、省级环保模范城市。志丹县、富县分别获得全国文明县城、国家卫生县城殊荣。黄陵县被列为省级新型城镇化试点县。文安驿镇、店头镇被列入全国第二批特色小镇，黄龙石堡镇、宜川云岩镇等17个镇被列为全国重点镇。城镇化率达到60%，较上年提高0.91个百分点。

（五）环境保护和生态建设力度持续加大。以中省环保督察整改为抓手，着力解决突出环境问题。全面落实河长制，编制4条主要河流水体达标方案，建成河流拦污设施2903个。节能减排任务全面完成，单位GDP能耗下降4%。城区空气质量优良天数达到313天，较上年增加23天。造林绿化88.1万亩，累计治沟造地38.2万亩。桥北省级森林公园晋升为子午岭国家森林公园。治理水土流失面积1010平方公里。乾坤湾成功创建国家水利风景区。农村人居环境整治工作走在全省前列，延川县梁家河村被评为陕西唯一的“中国最美乡村”，15个村被评为全省美丽宜居示范村，完成22个省级“三化一片林”绿色家园和50个贫困村美丽村庄建设，创建美丽干线公路269公里。

（六）改革开放深入推进。打好“三去一降一补”攻坚战，完成46户市属国有企业改制，启动41户国有企业“三供一业”分离移交。引导退出2处煤矿，淘汰落后产能45万吨。商品住房库存面积下降24.3%。减少涉企收费项目31项，为企业减负2700多万元。电力直接交易29.5亿千瓦时。完成农村土地承包经营权确权登记颁证工作，安塞南沟村、黄陵索洛湾村等一批“三变”改革典型备受关注。国有林场改革主体任务完成。水务一体化改革顺利推进。对外开放不断扩大，与遵义市、沈阳市、佛山市、美国堪萨斯市缔结友好城市。“招商季”活动成果丰硕，与北控、腾讯、中集等企业签订15个战略协议,比亚迪、万达等200多家企业来我市考察洽谈，实施招商引资项目630个，到位资金862.2亿元、增长22.2%，利用外资1553万美元。

（七）脱贫攻坚取得阶段性成果。举全市之力抓脱贫，市财政落实专项扶贫资金5亿元、增长1.5倍。苏陕扶贫协作实施项目23个。与省国企“合力团”共同发起成立延安产业扶贫公司，完成产业扶贫投资14.1亿元。“八个一批”脱贫工程扎实推进，实现有劳动能力贫困人口产业全覆盖，全国农业产业扶贫精准脱贫经验交流会在我市召开。争取光伏扶贫指标266.291兆瓦，1.6万贫困户连续20年每户每年稳定增收3000元。一次性启动建档立卡贫困人口易地扶贫搬迁，搬迁入住1.3万户4.55万人。农村危房改造走在全省前列。兜底保障实现全覆盖。维修改造92个贫困村“油返砂”公路366公里，532个标准化卫生室、1314处农村安全饮水设施全部建成，贫困村电力入户率达到100%。1.47万贫困人口实现脱贫，216个贫困村退出，延长县有望“摘帽”。

（八）人民群众生活持续改善。财政民生支出288.4亿元，占财政支出的81.7%。新增城镇就业1.65万人，城镇登记失业率为3.45%。实现城乡居民医疗保险、大病保险全覆盖和医保异地就医直接结算。普惠性幼儿园占比达到83%。子长、甘泉、洛川通过国家义务教育发展基本均衡县认定。创建全国足球特色学校72所，为全市中小学生免费发放足球3万个。延安市第一中学和宜川中学成功创建省级示范高中。延安大学新校区、市中医医院迁建工程、市精神病医院加快建设。市人民医院跻身三甲行列，第二医疗集团完成组建。加快创建健康城市和国家食品安全示范城市。完成29个乡镇综合文体中心、401个村（社区）综合文化服务中心新建扩建工程。宝塔区芦山峁超大型史前遗址考古取得重大发现。成功举办“奔跑中国·红色之旅”国际半程马拉松赛、中国·秦直道第三届自行车联赛、国际雪联越野滑雪中国巡回赛等8项重大赛事，全市经常参加体育锻炼人数达到75万人。安全生产“四项统计指标”全面下降，市安监局荣获全国安全监管监察工作先进集体等两项殊荣。积极排查矛盾纠纷，赴省进京上访总量分别下降8.2%和69.3%，中省重要会议活动期间实现“零非访”。扎实推进平安延安建设，群众社会治安满意度稳步提升，荣获全国社会治安综合治理最高荣誉“长安杯”。

（九）政府自身建设不断加强。认真学习贯彻党的十九大精神，扎实推进“两学一做”学习教育常态化制度化。加强党风廉政建设，严格落实中央八项规定精神。启动相对集中行政许可权改革试点，市行政审批服务局挂牌成立。行政许可事项办理时间平均压缩57.3%。公共资源交易中心平台建设和数据传送及时率、准确率走在全省前列。从严控制一般性支出，“三公”经费下降7%。自觉接受人大、政协和社会各界监督，办理人大代表建议117件、政协委员提案432件。网民留言办结率达到100%。“12345”智慧政务服务和社情民意调查平台上线运行。省级目标责任考核群众满意度调查位列全省第三，增幅全省第一。

统计、物价、人防、双拥、盐务、气象、税务、档案、方志、慈善、老龄、助残、民族宗教、妇女儿童、外事侨务、国家安全、国防动员、驻延部队和民兵预备役建设等工作都取得了新成绩。

各位代表，在转型发展、爬坡过坎的艰难时期，我市经济增长好于预期目标，摆脱了多年低速徘徊的被动局面。这是市委正确领导的结果，是市人大常委会、市政协监督支持的结果，是全市人民团结拼搏的结果。在此，我代表市人民政府，向全市干部群众，向各民主党派、工商联、无党派人士和离退休老干部，向中省驻延企事业单位、驻延部队、武警官兵和公安民警，向各位代表、政协委员和社会各界人士表示崇高的敬意和衷心的感谢！

在看到成绩的同时，我们也清醒地认识到，发展中仍面临一些突出矛盾和问题，主要有：经济结构不平衡，服务业仍然是短板，传统工业转型缓慢，新动能支撑作用还未显现；城乡发展不平衡，县域经济规模小，精准脱贫任务艰巨，基础设施保障和公共服务均等化水平不高；市场活力不充分，营商环境不优，民营企业数量少、实力弱，非公经济占比低；绿色发展不充分，生态环境依然脆弱，水、大气、土壤等重点领域污染防治形势严峻；社会治理创新不充分，财政金融领域风险潜存，社会稳定风险较大；部分干部思想观念、工作作风、责任担当与新时代要求不相适应。问题导向就是努力方向，我们将以抓铁有痕、踏石留印的韧劲，认真加以解决。

二、2018年经济社会发展预期目标和主要任务

今年是贯彻党的十九大精神的开局之年，是改革开放40周年，是决胜全面建成小康社会、实施“十三五”规划承上启下的关键一年。我们将以习近平新时代中国特色社会主义思想为指导，认真学习贯彻落实中央经济工作会议、省委十三届二次全会和市委五届四次全会精神，坚持稳中求进工作总基调，全力打好精准脱贫、污染防治、防范化解重大风险三大攻坚战，统筹做好经济社会发展各项工作。综合考虑当前和长远、速度和质量等因素，建议主要预期目标是：生产总值增长8%左右，全社会固定资产投资增长10%左右，地方财政收入增长8%，城乡居民人均可支配收入分别增长8.5%和9%左右。

实现上述目标，重点抓好十个方面工作任务：

（一）继续加快经济结构调整，推动经济高质量发展

深入实施创新驱动发展战略。加快科技资源统筹中心、孵化基地、创新空间建设，打造“延安·红色筑梦”创业创新品牌，让群众创业激情竞相迸发，把“有中生新、无中生有”两篇文章做得更加具体扎实，努力建设创新型城市。推动高新区晋升国家级高新技术产业开发区，支持安塞、志丹工业集中区建成省级高新技术产业开发区。建立科技创新基金，引导市场主体加大科研投入，推动科技创新与实体经济深度融合，实现量增质更优的发展。与高等院校、科研机构联合组建工程技术研究中心、产业技术联盟、重点实验室和院士工作站，推动集成创新和科研成果转化。培育数字经济、共享经济等新业态新模式，推动“互联网+”、云计算、大数据、物联网与先进制造业、现代服务业、文化旅游、教育医疗等行业领域跨界融合。完善新经济考核奖励办法，支持落地企业加快发展，引进新经济企业200家以上，实现营业收入60亿元以上。实施质量强市战略，开展质量提升行动，新注册商标2000件，打造一批知名商标和特色品牌。

加快建设延安综合能源基地。实施《延安综合能源基地发展规划》，建成富县煤油气资源综合利用、洛川轻烃裂解综合利用、安塞LNG等项目，加快延长石油伴生气资源循环利用、子长500万吨低阶煤多联产循环综合利用、宝塔区天然气综合利用、延川20万吨可发性聚苯乙烯、吴起20万吨LNG等项目建设。建成陕北至关中750千伏第二通道，争取延安至西柏坡电力外送通道纳入国家规划，推进陕北至武汉输电工程前期工作，加快华能延安电厂、洛川琦泉生物质热电联产和延安新区、安塞区多能互补等项目建设，力争开工黄陵电厂、富县电厂。建设新能源项目13个，实施第二批光伏扶贫项目，推动大唐热电联产2号机组并网运行。全年生产原油1590万吨、加工原油960万吨，生产煤炭5300万吨、天然气50亿立方米，LNG产量达到90万吨，发电50亿千瓦时。

全面振兴实体经济。培育壮大现代化工、装备制造、新材料、新一代信息技术、消费品工业五大产业，实施智能制造、绿色制造、质量品牌、技术进步、生产性服务和中小企业培育六大工程，重塑延安现代工业体系。创新高新区管理体制，发挥武汉中电光谷全生命周期软性服务功能，集聚生产要素和高端科技，推动新能源汽车、太阳能发电、绿色建材、医药等项目向产业链延伸，建成彩虹光伏玻璃、金风风机制造、华为云计算数据中心等项目，加快新加坡三达膜科技、弗莱茵机电科技等高新技术产业项目建设。继续提高中高端卷烟产品比重，加大地产卷烟营销力度，生产卷烟40万箱。支持延安制药、延安隆基药业发展，培育壮大医药产业。扶持建筑业骨干企业发展，组建市级建工集团，推动建筑行业转型升级，建筑业增加值增长8%。

全力支持民营企业发展。深化商事制度改革，全面推行“证照分离”、“多证合一”，实现“准入即营”，新增中小微企业7000户。设立中小微企业扶持基金，完善融资担保体系，组建西北金融控股集团，盘活金融资源，着力破解民营企业融资难、融资贵问题。加大上市企业培育力度，推进延安企业在陕西股权交易中心和新三板挂牌，培育“顶天立地”的排头兵企业和有影响力的企业家队伍。完善市场准入负面清单，清理规范涉企收费，鼓励非公经济进入能源转化、高新技术、节能环保等领域。

（二）大力实施乡村振兴战略，加快推进农业农村现代化

坚持农业农村优先发展。认真贯彻“中央1号文件”精神，按照“产业兴旺、生态宜居、乡风文明、治理有效、生活富裕”的总要求，尊重乡村发展规律，科学制定乡村振兴规划。加大涉农资金整合力度，对行业内涉农资金在预算编制环节进行源头整合，行业间涉农资金在预算执行环节进行统筹，支持乡村振兴事业。强化金融支农力度，完善农村金融服务体系，更好满足乡村振兴多样化金融需求。加强乡村人力资源开发，扶持培养一批职业经理人、经纪人、文化能人和非遗传人。清除各类要素下乡障碍，吸引资金、技术、人才等向农村流动。顺应农村人口向城镇转移趋势，合理调整乡村布局，加快编制村庄建设发展规划，行政村规划编制覆盖率达到80%以上。统筹城乡基础设施建设和产业配套，增强农村发展后劲，推进城乡融合发展。

加快构建现代农业体系。坚持稳粮、优果、兴牧、扩蔬，深化农业供给侧结构性改革，推动农业由增产向提质转变。推进国家现代农业示范区建设，新启动市级现代农业示范园20个，建成13个。培育壮大新型农业经营主体，扶持发展“一村一品、一乡一业”专业镇16个、专业村210个。做优做强苹果产业，支持中国洛川苹果科技创新中心建设，加快土壤改良、品种换代和老旧园改造，全面提升“洛川苹果”、“延安苹果”、“梁家河苹果”品牌影响力。促进标准化规模化养殖，持续扩大设施蔬菜种植面积，大力支持食用菌、中药材及花卉苗木、油料等特色种养业发展。全力推进以苹果为主的特色农业产业后整理，给特色农产品穿上文化的马甲，插上互联网的翅膀，促进农民全产业链增收。大力发展林下经济、休闲农业和农村电商等新产业新业态，促进农村一二三产业融合发展。推进“三品一标”认证和农业标准化生产，强化农产品质量安全全程监管。加强防灾减灾、技术服务、疫病防控、农资监管体系建设，提升农业生产机械化水平，拓宽政策性农业保险覆盖面，加快构建现代农业产业体系、生产体系、经营体系。

深化农村各项改革。坚持以完善农村产权制度和要素市场化配置为重点，落实农村土地承包延期政策，抓好30个农村产权制度改革和宝塔、甘泉、富县“三权分置”试点，继续推进农村“三变”改革。在依法保护集体土地所有权和农户承包权前提下，平等保护土地经营权，促进要素更多流向农业。采取土地转包、出租、托管、代耕、股份合作等方式，有效流转农村土地。建立集体资产管理制度，加快清产核资、成员界定、股权量化、经营性资产量化改革，实现规模经营，增加集体收入，带动农民发展。建立市县乡三级农村产权交易服务体系，引导土地经营权、集体林权、“四荒”地使用权规范流转交易。

提升乡村宜居质量。巩固农村人居环境综合治理成果，狠抓农村生活垃圾、污水处理和卫生改厕，推广垃圾“户分类、村收集、镇运转、县（镇）处理”和“垃圾兑换银行”模式，持续整治公共空间和庭院环境，消除脏乱差。大力提升农村建筑风貌，开展田园建筑示范，建设绿色生态村庄，将所有村建成清洁村，再有10%的村建成美丽宜居示范村、30%的村建成生态村。完善村民自治机制和村规民约，提升农民综合素质和乡村文明程度。倡导移风易俗，坚决遏制大操大办、厚葬薄养、奢侈浪费、人情攀比等陈规陋习，树立良好风尚，建设美丽乡村、文明家园。

（三）加快文化旅游产业发展，促进现代服务业提档升级

构建全域旅游新格局。完成全域旅游发展规划编制，推动旅游资源共享，抓好宜川、黄陵首批国家全域旅游示范区和延川、黄龙第三批省级旅游示范县创建。加快黄帝陵国家文化公园、中国革命文艺家博物院、延安“红谷”丹霞地质遗迹景观走廊、安塞黄土风情园和文化旅游名镇、文化古镇建设，推进延安革命纪念地、壶口瀑布、乾坤湾、黄陵国家森林公园5A级景区创建。加快南泥湾红色文化小镇建设，完善道路、信息、商业等服务功能，再现“陕北好江南”。加大秦直道、古长城、石窟群等历史遗址保护开发力度，推出经典历史文化旅游产品。打造一批乡村旅游示范村，建设研学旅行、红色教育培训、体验式教学和森林康养基地。

提升文化旅游产业发展水平。建成宝塔山、枣园、杨家岭、西北局游客服务中心和宜川、黄陵、延川、黄龙游客集散中心，加快星级酒店群、旅游厕所、景区停车场和智慧旅游应急指挥平台建设。精心打造延安特色小吃品牌，扶持发展清真餐厅，满足游客饮食消费需求。开发文化创意产品，丰富旅游商品市场。加强旅游市场管理，制定行业标准，提升游客满意度。策划青年筑梦、寻根谒祖、红色记忆、自然观光等精品旅游线路，放大“十佳魅力城市”品牌效应，深度策划优秀旅游城市形象宣传。做大做强红色教育培训产业。抓好六大文化产业园区和24个县级重点文化产业项目建设，文化产业增加值增长13%。

促进现代服务业发展。完善电商服务网络，构建四级物流配送体系，支持和规范快递业发展，促进特色产品线上线下销售。加快17个现代物流园区、义乌智慧商城和上海新城控股商业综合体建设，支持利源物流发展城市零担货运。加强与山东卓创、上海讪叶等企业合作，发展大宗商品贸易。进出口贸易总额增长12%。推动连锁品牌企业设立社区便利店、连锁店和生鲜超市，打造“15分钟”社区便民服务圈。社会消费品零售总额增长10%。加大“五上”企业培育力度，推进在延企业注册入统和集中统一结算。第三产业增加值增长11%。

（四）发挥区位和资源优势，加速释放区域经济发展新活力

壮大县域经济实力。落实支持加快县域经济发展的意见，采取以奖代补措施，推动县域经济差异化、特色化发展，建设“现代农业强县”、“新型工业强县”和“生态建设强县”，力争1个县进入全省“十强县”，其他县区排名位次明显前移。加快县域工业集中区基础设施建设，促进更多企业和项目落地，提高园区对县域经济的贡献率。依托优势产业和骨干企业，发展科技含量高、资源消耗少、环境污染小、经济效益好的新型产业园区，推动县域经济向集约集群、高端链条化方向发展。

推动沿黄经济带建设。制定沿黄经济带区域发展总体规划和生态城镇带建设三年行动计划，依托沿黄公路和黄河沿岸自然景观、历史文化、特色资源优势，统筹推进城镇建设、产业发展、生态保护和人居环境整治，建设旅游观光带、产业示范带和生态城镇带，带动沿线群众脱贫致富。

推进延塞一体化发展。制定延塞一体化发展规划，启动延安新动能产业园区建设，布局重大产业项目，建设延塞经济走廊。加快延塞快速干道建设，推进中心城区与安塞区在基础设施、公共服务等方面共建共享、功能互补，打造延塞半小时经济圈，形成城市经济增长新板块。

（五）狠抓重大项目建设，努力促进固定资产投资增长

千方百计扩大固定资产投资。坚持重大项目集中审批，落实环境保障属地责任，推动政府从“抓项目”向“抓环境”转变。完善政府和社会资本合作模式，规范推进PPP项目落地。发挥政府投资引导作用，鼓励民间资本进入基础设施和公共服务领域，扩大民间投资规模。制定招商引资奖励办法，围绕能源化工、新型建材、新能源汽车、文化旅游、现代农业、新经济六大产业，开展精准招商，全年引进50个投资2亿元以上的生产性产业项目。

加快改善基础设施条件。建成延安新机场和吴起至定边高速公路，加快西延高铁、蒙华铁路以及延长至黄龙等7条通县高速公路建设，推进龙安水库、永宁山水库项目前期。加强乡村公路养护，实现100%行政村通沥青路。开工建设330千伏延安东输变电工程。升级延安至西安宽带网络。开展第三批电信普遍服务试点，完成535个行政村建设任务。

加强项目谋划争取。密切与央企省企、高等院校、科研机构合作，紧盯国家推进“一带一路”建设、供给侧结构性改革、脱贫攻坚等政策导向，研究策划重大产业、基础设施和公共服务项目。发挥延安政治优势，积极争取中省支持，力争更多项目进入中省计划盘子。加大项目前期论证投入，成立重大项目研究机构，谋划、储备、培育、实施一批能够支撑带动全市经济社会发展的大项目、好项目。

（六）加快新型城镇化步伐，彰显革命圣地新形象

深入开展城市修补生态修复。出台延安“城市双修”总体规划，坚持“疏密、拆旧、补缺、建新、提质”相结合，避免大拆大建，留住和恢复更多“老延安”记忆。加快推进棚户区改造，全面提升城市公共服务功能。编制城市综合交通规划，逐步打通城市环线和“断头路”。优先发展公共交通，规划实施城市旅游轻轨。开展国家生态园林城市、国家节水型城市创建，拓展绿色空间，实施城市排水防涝补短板三年行动计划。争取将延安宝塔、黄帝陵纳入全国历史建筑名录，枣园、杨家岭、鲁艺等街区纳入全国历史文化街区名录。做好县城“双修”规划编制和重点街区城市设计，开展县城“双修”。

加快城镇体系建设。全面推进新区、高新区、南泥湾景区“多规合一”、“多评合一”。加快新区市政基础、学校医院、商贸物流、运动休闲等设施建设，导入大数据、金融服务、酒店餐饮、综合商业等业态，完善服务功能，提高房屋入住率，实现产城融合，增强城市活力。创建一批省级示范县城，打造市域副中心城市。抓好国家特色小镇和省市县重点镇建设，扶持发展一批产业强镇和综合型强镇。加强历史文化名村和传统村落保护。增强城镇对农业转移人口的吸引力和承载力，城镇化率达到61.5%。

全面提升城市治理水平。严格执行《城市规划导则》，持续开展“双违”整治，集中开展擅自改变规划用途、广告灯箱牌匾、杆线整理入地三项整治。认真落实《延安市城市市容市貌管理条例》，全面推进新型智慧城市建设，提高城市管理法治化、规范化、精细化水平。理顺物业管理体制，规范物业市场秩序，加快老旧小区水电气暖等设施改造，努力提高公共服务能力。

（七）凝聚强大合力，坚决打赢脱贫攻坚战

不折不扣完成脱贫攻坚任务。坚持以脱贫攻坚统揽经济社会发展全局，把脱贫攻坚作为头等大事和第一号民生工程，作为政治任务和发展机遇，紧盯“两不愁、三保障”目标，统筹推进“八个一批”脱贫工程，实施30户以上自然村“组组通”、安全饮水和自然村通动力电等基础设施“三提升”工程，补齐贫困村基础设施和公共服务短板，壮大农村集体经济。用好省域内城乡建设用地增减挂钩政策，完成4022户12452人易地扶贫移民搬迁。抓好农村危房改造。高质量完成3.7万贫困人口脱贫、338个贫困村退出和延川、宜川两个贫困县“摘帽”任务，在全国革命老区中率先实现整体脱贫。

全面推进产业扶贫。把产业扶贫作为“拔穷根”的根本举措，坚持发展短平快项目与培育特色产业相结合，优先落实产业扶持政策，完善生产奖补措施，依托资源禀赋，因户而异培育特色产业，确保贫困群众通过发展产业实现稳固脱贫。发挥农业龙头企业、农民专业合作社作用，建立新型经营主体与贫困村、贫困户利益联结和脱贫带动机制，引导帮助贫困群众发展产业。支持信合利用自身优势，加大扶贫小额贷款投放力度。坚持扶贫与扶志、扶智相结合，广泛开展技能培训，拓宽就业渠道，提高脱贫能力，增强贫困群众内生动力。

强化精准脱贫保障措施。坚持“八办两组”协调联动、“四支队伍”驻村联户工作机制，强化延安与无锡扶贫协作，争取省国企“合力团”支持，巩固大扶贫格局。市县财政扶贫资金不低于地方财政收入计划的2%，增幅不低于20%。开展扶贫资金绩效管理，提高扶贫资金使用效益。对照“477”标准，下功夫抓好脱贫问题整改。定期核查建档立卡信息，做好贫困人口信息动态调整，完善精准扶贫台账，推进数据共享，打造智慧扶贫新模式。加强扶贫领域腐败和作风专项治理，严肃查处弄虚作假、数字脱贫行为。坚持攻坚期内脱贫不脱政策，健全脱贫长效机制。

（八）坚决打好污染防治攻坚战，全面提升环境保护和生态文明建设水平

着力解决突出环境保护问题。继续加大中省环保督察交办问题整改力度。开展第二次全国污染源普查。坚持重拳治水、“一河一策”，全面落实河长制，实施水体达标行动。建成市第二污水处理厂，规范县级污水处理厂运行，加快重点镇和河流沿线城镇污水处理厂建设，提高污水收集率和处理率，强化输油管道安全监管，提升水环境质量。坚持铁腕治霾，建立市区、市县联防联控机制，打好“减煤、控车、抑尘、治源、禁燃、增绿”组合拳，确保城区空气质量优良天数达到300天以上，进一步增强群众蓝天幸福感。开展净土行动，启动土壤污染详查和治理，严控土壤面源污染风险。

加快建设美丽延安。推进国家生态文明先行示范区建设，实施主体功能区规划，完成生态红线划定，制定生态文明建设目标评价考核办法，全面推开自然资源资产负债表编制试点，扎实开展领导干部自然资源资产离任审计。提高国家森林城市建设水平，推进“陕北高原大绿化”，加快1000万亩常青生态林建设，完成造林50万亩。抓好国家和省级森林公园群建设，推进桥山、黄河国家公园前期工作，逐步实现13个县区森林公园全覆盖。加快城乡居民居住区和县域工业集中区园林绿化，打造三季有花、四季常青、全年是景的“大花园”。坚持封山禁牧不动摇，巩固提升退耕还林成果。加快延河综合治理和黄河西岸生态整治，系统推进山水林田湖草综合治理。治理水土流失1000平方公里，完成治沟造地12.5万亩，新修改造基本农田9万亩。

全面推进节能减排。实行排污许可“一证式”管理，落实环境影响评价、排污许可、环境监测、环境信息公开、污染物排放总量控制等制度，抓好重点能耗企业落后产能淘汰和节能新技术、新工艺推广应用，确保达标排放。依法开展项目节能评估审查，落实节能降耗措施，单位GDP能耗下降3.2%，发展绿色、节能和装配式建筑，绿色建筑占新建建筑比例达到40%以上。

（九）深化改革扩大开放，加快构筑发展新高地

进一步简政放权。实施“营商环境提升年”行动，深化“放管服”改革，从严控制新增审批事项，规范中介服务，开展县域营商环境监测评价，实现“双随机、一公开”监管全覆盖。推进相对集中行政许可权改革试点，构建“流程最优、时限最短、成本最低、服务最好”的行政审批机制。上半年运行“互联网+政务服务+人工智能”平台，推行市级行政审批全程电子化、网上办、“不见面”，年底前实现80%的行政许可事项网上审批。

深化重点领域改革。打好防范化解重大风险攻坚战，加强政府性债务风险管控和融资平台监管，严格控制增量，逐步消化存量，强化金融风险监测和应急处置，严厉打击违法违规金融活动，守住不发生系统性金融风险底线。深化国企国资改革，抓好“僵尸企业”处置，推进市属国有企业股份制、公司化改革，发展混合所有制经济。完成37户国有企业社会职能移交和39户改制企业“三供一业”分离移交。实行市属经营性国有资产集中统一监管，确保国有资产保值增值。推进财政事权和支出责任划分改革，深化预决算公开和绩效预算管理。统筹抓好水务一体化、国有林场、供销社、盐业体制等各项改革。

全方位扩大对外开放。抓住国家加大西部开放力度的机遇，依托“大西安”，主动融入“一带一路”建设，争取延安航空口岸开放，发挥立体交通优势和延安海关、国检、边检等口岸联检机构作用，以高新区为平台，开展自贸区试点，扩大国际经贸合作交流，发展枢纽经济、门户经济、流动经济。推动与阿拉善盟、南昌市、大连市缔结友好地市，加强与友好城市、沿海沿边发达地区在产业、科技、金融等方面合作，建设友好城市产业转移基地。发挥第三届丝博会“主题市”优势，推动与中亚国家、“一带一路”沿线城市展开务实合作。

（十）优先保障和改善民生，不断提升人民群众幸福感获得感安全感

努力提升就业质量。继续实施促进创业带动就业五年行动计划，落实创业担保贷款政策，扶持重点人群自主创业，释放创业带动就业潜力。支持延安职业技术学院与企业合作，实施“订单式”培养、“委培式”代训、“定向式”招聘，改善劳动力结构，有效扩大就业。引导高校毕业生转变择业观念，鼓励到中小企业就业。新增城镇就业1.5万人，城镇登记失业率控制在3.9%以内。引进高层次紧缺型人才，实施教育、卫生“百千万”人才培养工程，解决高精尖人才短缺和基层教育卫生力量薄弱问题。

健全完善社会保障体系。坚持量力而行、尽力而为，全面实施全民参保计划，完善社会保险制度。加快社区养老日间照料中心、农村互助幸福院建设和市社会福利院迁建，完成儿童福利院改造。健全残障人员、留守儿童、弱势群体工作网络，实施精准助残服务，维护特殊困难群体合法权益。积极推进土地利用开发，有效增加住房供给，大力发展住房租赁市场，扩大住房公积金覆盖面，建立多主体供给、多渠道保障、租购并举的住房制度。

优先发展教育事业。完善校际联盟和大学区制管理，加快改善城乡办学条件，以信息化推动优质教育资源共享，着力解决城区上学入园难、大班额、择校热以及婴幼儿照护、儿童早期教育服务、中小学生课外负担重等突出问题。实施第三期学前教育行动计划和城区初中教育质量提升工程。宝塔区通过国家义务教育发展基本均衡县评估认定，甘泉县、宝塔区通过省政府“双高双普”验收。加强师德师能考核，实现义务教育学校视导全覆盖，提高教育教学质量。大力发展校园足球运动。启动延安市特殊教育学校建设。加快延安大学新校区建设。支持延安职业技术学院创建全国优质专科高职院校。办好网络教育和继续教育。

加快向健康城市迈进。加大城市公立医院改革力度，深化县级公立医院改革。推动第二医疗集团实质性运行，逐步实现区域内分级诊疗和市域内二级以上医院检查结果互认，减轻群众看病负担。广泛开展健康教育，提高居民健康素养。加强传染病和慢性病综合防控，倡导健康生活方式。落实两孩政策，提高出生人口素质。巩固国家卫生城市创建成果，全面开展健康城市建设。抓好国家食品安全示范城市创建，强化“四品一械”监管，落实“四有两责”，提高食品药品安全保障水平。

推动文体事业快速发展。推进国家级陕北文化生态保护实验区建设。加快筹建全国文化干部培训实践基地。建立现代公共文化服务体系，着力繁荣文化艺术创作。完成37个乡镇综合文体中心、396个村（社区）综合文化服务中心建设，基层综合性文化服务中心年度达标率达到20%。建成开放市图书馆。启动延安博物馆、新闻传媒中心建设。加快第十四届全运会比赛场馆建设，广泛开展全民健身活动。

加强社会治理创新。继续推进全国文明城市创建。做好第十次村委会换届选举工作。加强城乡社区标准化建设。扎实做好初信初访、网络信访和积案化解工作。深化安全生产领域改革发展，加强重点领域安全生产监管。深入开展扫黑除恶专项斗争，严厉打击涉黑涉恶违法犯罪。坚持失信联合惩戒，推动信用“红黑榜”常态化发布。规范民间信仰事务管理，依法提高民族宗教工作水平。完善应急管理体系，提升应急处置能力。扎实做好双拥和国防动员工作，大力支持军队和预备役建设。

各位代表，“增进民生福祉是发展的根本目的。”今年，我们将重点办好“民生十件实事”：一是实施棚户区改造3.2万套；二是改造老旧小区5个；三是新建扩建市区学校15所、幼儿园12所；四是完成市中医医院迁建和精神病专科医院建设，启动新区妇女儿童医院建设；五是建设延安新区全民健身运动中心；六是新建、改扩建社区卫生服务中心9个；七是实施便民服务工程，建设一批城市便利加油站、果菜超市和停车场（楼）；八是实施畅通延安工程，建设城市道路连接线5条；九是推进“厕所革命”，新建改造旅游厕所95座、城区公厕63座、县城公厕108座；十是分区域建设3个殡葬吊唁场所。

三、努力建设人民满意的政府

新时代、新征程对政府工作提出新要求，必须加快转变政府职能，提高政府治理能力和水平。

提高学习能力，增强履职尽责本领。深入学习习近平新时代中国特色社会主义思想，用伟大思想武装头脑，做到信念过硬、政治过硬、责任过硬、能力过硬、作风过硬。坚持“两学一做”学习教育常态化制度化，扎实开展“不忘初心、牢记使命”主题教育，加强学习，不断汲取智慧、增长才干、增强本领。坚定改革定力，大胆解放思想，创新工作方式，努力创造无愧于圣地人民的新业绩。

坚持依法行政，着力建设法治政府。把法治理念贯穿政府工作全过程，创建法治政府示范市。普遍建立法律顾问制度，坚持市政府常务会议集体学法和领导干部法律学习制度。完善科学民主依法决策制度，把公众参与、专家论证、风险评估、合法性审查、集体讨论决定作为重大决策必经程序。落实“谁执法谁普法”责任，深入实施“七五”普法。自觉接受市人大及其常委会法律监督、市政协民主监督和社会公众监督，认真办理人大代表建议和政协委员提案。

持续转变作风，着力建设高效政府。加大政务公开力度，开展“减证便民”专项行动，实行证明事项和盖章环节清单管理，推进政务数据资源共享，打通服务群众“最后一公里”。坚持干字当头，争做行动派实干家，抓住群众最急最忧最怨问题精准发力，以功成不必在我的胸怀、以“钉钉子”精神推动延安发展。完善重点工作通报讲评制度，奋勇争先进位，做好“四问”答卷。坚持正向激励、反向倒逼，严格落实“三项机制”，为干部干事创业撑腰鼓劲。

严格正风肃纪，着力建设廉洁政府。深入推进政府系统全面从严治党工作，严格落实中央八项规定实施细则和市委实施办法，驰而不息纠正“四风”，严肃问责表态多调门高、行动少落实差问题。坚持用制度管权管事管人，扎紧织密制度“笼子”。严格财经纪律，严控“三公”经费，强化审计监督。坚持无禁区、全覆盖、零容忍，严厉整治发生在群众身边的腐败问题，构建“亲”“清”政商关系，以实际行动赢得群众信任和支持。

各位代表，“幸福是奋斗出来的”，只有奋斗才能创造美好生活。让我们更加紧密地团结在以习近平同志为核心的党中央周围，以习近平新时代中国特色社会主义思想为指导，在市委的坚强领导下，不忘初心，牢记使命，低调务实，埋头苦干，为决胜全面建成小康社会、不断满足延安人民日益增长的美好生活需要而努力奋斗！
